# Compatibility of Carbonate Mixtures to Be Used as Molten Salts with Different Metal Alloys to Be Used as Container Materials

**DOI:** 10.3390/ma18071541

**Published:** 2025-03-28

**Authors:** Luisa F. Cabeza, Franklin R. Martínez, Emiliano Borri

**Affiliations:** GREiA Research Group, University of Lleida, Pere de Cabera 3, 25001 Lleida, Spain; rodrigo.martinez@udl.cat (F.R.M.); emiliano.borri@udl.cat (E.B.)

**Keywords:** molten carbonate salts, corrosion, high-temperature applications, solar energy, carbon dioxide capture and storage (CCS), metal alloys

## Abstract

The energy transition can only be achieved if the global energy sector is transformed from a fossil-based system to a zero-carbon-based source system. To achieve this aim, two technologies have shown promising advances in high-temperature application. Concentrating solar power (CSP) plants are seen as a key technology to achieve the needed energy transition, and carbon dioxide (CO_2_) capture and storage (CCS) is a promising technology for decarbonizing the industrial sector. To implement both technologies, molten carbonate salts are considered promising material. However, their corrosive behavior needs to be evaluated, especially at high temperatures, where corrosion is more aggressive in metal structures. This paper presents an experimental evaluation of the static corrosion of two molten carbonate salts, a Li_2_CO_3_-Na_2_CO_3_-K_2_CO_3_-LiOH∙H_2_O (56.65-12.19-26.66-4.51wt.%) mixture and a Li_2_CO_3_ salt, under an air atmosphere with five corrosion-resistant metal alloys, including Alloy 600, Alloy 601, Alloy 625, Alloy 214, and Alloy X1. In this study, the corrosion rate and mass losses were quantified. In addition, in all the cases, the results of the experimental evaluation showed corrosion rate values between 0.0009 mg/cm^2^·yr and 0.0089 mg/cm^2^·yr.

## 1. Introduction

As early as 2005, the Intergovernmental Panel on Climate Change (IPCC) highlighted the potential of carbon dioxide (CO_2_) capture and storage (CCS) as an option in the portfolio of mitigation actions for the stabilization of atmospheric greenhouse gas concentrations [1]. CCS is a process consisting of the separation of CO_2_ from industrial and energy-related sources, transport to a storage location, and long-term isolation from the atmosphere. Other mitigation options include energy efficiency improvements, the switch to less carbon-intensive fuels, nuclear power, renewable energy sources, enhancement of biological sinks, and reduction in non-CO_2_ greenhouse gas emissions. It is well known that given the intermittent nature of most renewable energy sources, thermal energy storage (TES) is a key technology for their deployment [2]. Both technologies, key to the success of the energy transition, have in common that they use molten salts, and more specifically, molten carbonates.

### 1.1. Use of Carbonates in CCS

**Figure 1:**
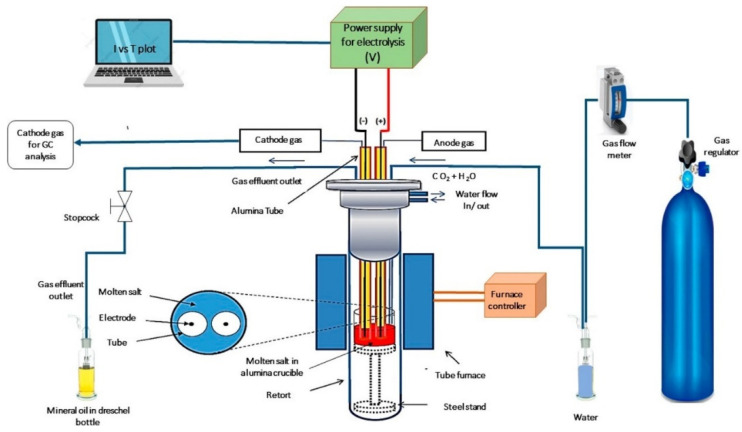
Experimental set-up to develop the concept of CCS using molten carbonate [3].

An innovative concept for CCS is the use of molten salt electrolysis. This process was developed by capturing and converting CO_2_ into valuable products via electrodepositing solid carbon from CO_2_ into two mixtures, a known eutectic mixture of Li_2_CO_3_, Na_2_CO_3_, and K_2_CO_3_ and a new mixture containing 0.1 mol of LiOH in addition [3,4]. This concept is based on bifunctional oxygen reduction reaction (ORR) and hydrogen evolution reaction (HER) catalysts, directly derived from CO_2_, in a process that captures carbon dioxide from the atmosphere or flue gases instead of producing it as all previous methods for creating ORR/HER catalysts. The experimental concept is presented in Figure 1. The electrochemical process is carried out in a cylindrical stainless steel reactor where the anode and cathode are placed. Holes are drilled in the flange cover to collect the gases from the electrode surface exposed to the molten salt.

### 1.2. Use of Carbonates in TES

**Figure 2:**
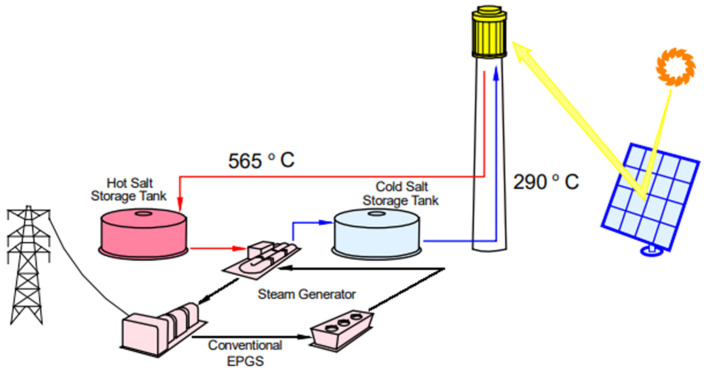
Schematic flow diagram of molten salt tower plant [7].

Concentrating solar power (CSP) plants use molten salts commercially as the storage media in their TES systems [5] (Figure 2), but these commercial plants use nitrate-based molten salts. With the purpose of increasing the temperature of the storage system to increase plants’ efficiency, other salts are being considered. Mixtures containing chloride salts are very interesting due to their low cost, but their use presents severe corrosion problems with potential materials for container tanks such as stainless steel. The mixtures of carbonate salts are an interesting alternative to chloride salts due to their lower corrosive potential. Carbonates possess a high heat capacity and high energy density and, therefore, require smaller tank volumes [6]. Within those studied carbonates, the molten eutectic ternary Li_2_CO_3_-Na_2_CO_3_-K_2_CO_3_ was also considered.

### 1.3. Literature Review on Use of Carbonates

A common issue that occurs with using molten salts and their high operating temperatures that can reach up to 700–900 °C is corrosion, which can challenge the long-term stability of electrodes [8]. Given the potentiality of molten carbonate mixtures in the two applications listed above, among others, this paper studies their corrosion behavior with more corrosion-resistant metals.

Several authors studied the corrosion performance of stainless steel- and Ni-based alloys in molten carbonate mixtures. This information is summarized in Table 1. A carbonate mixture more frequently studied is Na_2_CO_3_ + Li_2_CO_3_ + K_2_CO_3_, with different concentrations around the theoretical eutectic, such as 33.4wt.%Na_2_CO_3_ + 32.1wt.%Li_2_CO_3_ + 34.5wt.%K_2_CO_3_, 33wt.%Na_2_CO_3_ + 32wt.%Li_2_CO_3_ + 35wt.%K_2_CO_3_, and 34wt.%Na_2_CO_3_ + 33wt.%Li_2_CO_3_ + 33wt.%K_2_CO_3_, or with other concentrations, such as 35.1wt.%Na_2_CO_3_ + 10.2wt.%Li_2_CO_3_ + 54.5wt.%K_2_CO_3_, 31.2wt.%Na_2_CO_3_ + 15.5wt.%Li_2_CO_3_ + 53.3wt.%K_2_CO_3_, and 38wt.%Na_2_CO_3_ + 33wt.%Li_2_CO_3_ + 29wt.%K_2_CO_3_, in combination with both stainless steels (316, 321, 347) or more corrosion-resistant alloys such as In601, In800H, OC4, In626, etc. The corrosion rates measured at temperatures around 450 °C and 800 °C after exposure times between 24 h and 2000 h varied, but showed better resistance when Ni-based alloys were used compared to results with stainless steel. Similar conclusions were found with 28wt.%Li_2_CO_3_ + 72wt.%K_2_CO_3_ and with 59.4wt.%Na_2_CO_3_ + 40.6wt.%NaCl.

An experimental variation is testing under an inert atmosphere, which is possible to use in molten salt TES application in CSP plants, but not in ORR/HER catalysts for CCS. The tests of 47.19wt.%Na_2_CO_3_ + 52.81wt.%K_2_CO_3_ were not very good with the stainless-steel 316L but were similar when using stainless steel 347H or Inconel 800H. Therefore, when an inert atmosphere is possible, the use of some stainless steel alloys is possible, avoiding the use of more expensive Ni-based alloys.

### 1.4. Motivation of the Paper

This literature assessment shows that there are a lot of carbonate mixtures that have not been evaluated for corrosion resistance and that the consequences of gas formation in corrosion resistance have also not been evaluated. Therefore, this paper aims to evaluate two different carbonate-based molten salts considering both non-inert atmosphere and potential gas formation.

## 2. Materials and Methods

### 2.1. Materials

The molten salts evaluated were a Li_2_CO_3_-Na_2_CO_3_-K_2_CO_3_-LiOH∙H_2_O (56.65-12.19-26.66-4.51wt.%) mixture and a Li_2_CO_3_ salt. The chemicals used to prepare the mixtures were Li_2_CO_3_ (100% purity) from VWR, United States; K_2_CO_3_ (99.9% purity) from VWR, Germany; Na_2_CO_3_ (99.9% purity) from the United States; and LiOH·H_2_O (98% purity) from ThermoScientific, United States.

Due to their well-known high corrosivity with common metals like steel and stainless steel [13,17,19], other more corrosion-resistant metal alloys with high chromium and nickel content, available on the market and recommended as suitable materials to build containers for industrial applications, were used. The metal alloys selected were Alloy 600, Alloy 601, and Alloy 625 from ConecBand [22] (Tarragona, Spain), and Alloy 214 and Alloy X1 provided by UpCatalyst, partner of the project MoReCCU. The chemical composition of the metal alloys is shown in Table 2.

### 2.2. Methods

**Figure 3:**
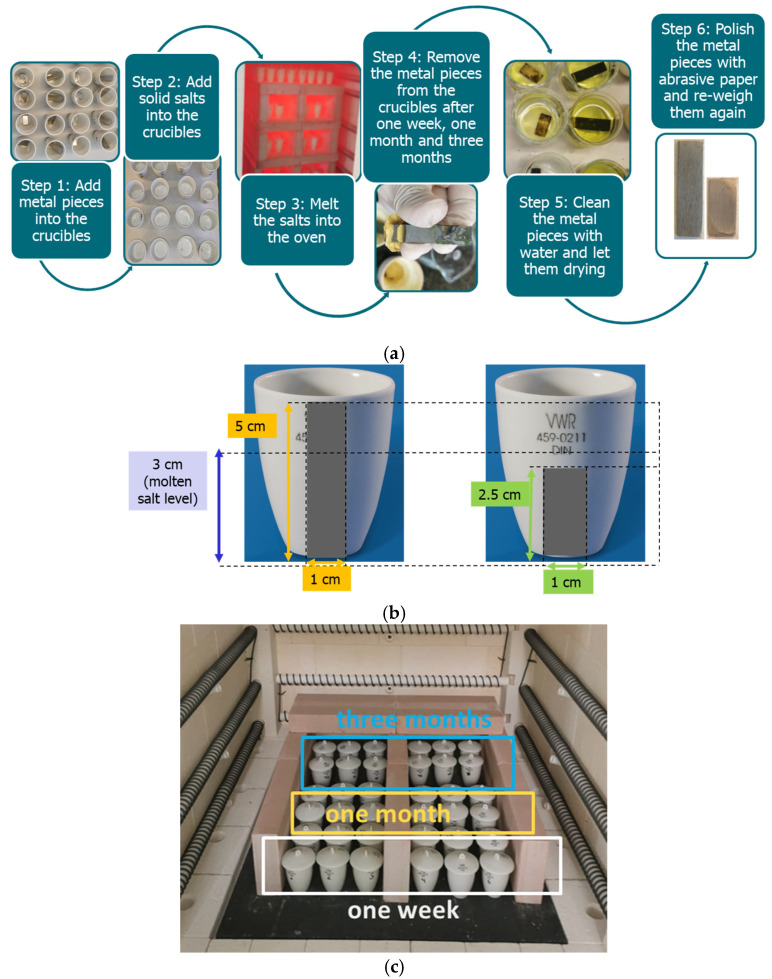
Methodology followed: (**a**) method steps, (**b**) metal alloy samples immersed in the experimental crucibles, and (**c**) samples in the oven.

The methodology used in the experimentation was as follows (Figure 3a) [23,24]. The metal alloys were cut and cleaned to remove any dirt and residue from prior use and the cutting process [25]. Then, the metal pieces were weighted in a precision balance AG135 from Mettler-Toledo with a precision of ±0.01 mg (Switzerland). The corrosion test used was a simple immersion test, but two different approaches were applied. In the first one, the metal samples were smaller (2.5 cm length), and they were completely immersed in the molten salt, while in the second one, the metal samples were longer (5 cm length) to ensure that half of the sample was immersed in the molten salt and the other half was not (Figure 3b). This was carried out to allow us to detect potential corrosion in the interface area [26]. Both the molten salt and the metal alloy were immersed in vitrified porcelain crucibles. Then, the crucibles were placed inside a Nabertherm model LH-216/12 electrical oven (Germany) at 780 °C (Figure 3c). The samples were removed from the oven after 1 week (7 days), 4 weeks (28 days), and 12 weeks (84 days), to evaluate the corrosion rate. In addition, three samples were assessed for each case (one sample and two duplicates), and the reported values of mass loss and corrosion rate are an average of these.

The evaluation of the metal alloy samples after testing started with a visual inspection, looking for bubbles, precipitates, surface changes, and potential pitting. Then, the metal pieces were cleaned, polished with abrasive paper if needed, and dried, followed by weighing them again.

The corrosion rate was calculated with the following formula:

$$CR=\frac{∆m}{A\cdot \left(t_{0}-t\right)}$$

where *CR* is the corrosion rate in mg/cm^2^·yr, *A* is the area in cm^2^, *t* is time in yr, and Δ*m* is the mass change calculated as follows:

$$∆m=m\left(t_{0}\right)-m\left(t\right)$$

where *m*(*t*_0_) is the mass at the beginning of the experimentation in mg and *m*(*t*) is the mass at the end in mg.

According to the literature [24], the industry only accepts corrosion rates lower than 0.2 mg/cm^2^·yr for long-term service, although corrosion rates between 0.3 and 9.9 mg/cm^2^·yr are acceptable. Corrosion rates higher than 10 mg/cm^2^·yr are not acceptable.

Moreover, the corrosion rate can also be expressed in mm/yr, and it is calculated as follows [6]:

$$CR=\frac{8760\cdot ∆m}{\rho \cdot t}$$

where *CR* is the corrosion rate in mm/yr, ∆m is the mass change per unit initial surface area in mg/cm^2^, ρ is the density of the material in g/cm^3^, t is the exposure time in h, and 8760 is the number of hours per year.

## 3. Results

Table 3 and Table 4 show the samples of metal alloys before and after testing and contact with both of the molten salts considered. Although there were some color changes in the samples, none of them were permanent, and corrosion-related changes were seen.

From the samples evaluated, it can be observed that all the alloys examined showed corrosion to the naked eye after immersion in Li_2_CO_3_-Na_2_CO_3_-K_2_CO_3_-LiOH∙H_2_O (56.65-12.19-26.66-4.51wt.%) and Li_2_CO_3_ during the experimental period. After being immersed in the molten salts, Alloy 600, Alloy 601, Alloy 625, Alloy 214, and Alloy X1 suffered a change in their appearance, with the samples taking different colorings, among which mainly dark coloring and brown spots related to the typical corrosion process can be distinguished [27].

The half-immersed samples showed greater deterioration in the interface than in the rest of the metal piece. This is in accordance with the expected results since the surface of the molten salt is in contact with a higher concentration of oxygen, a phenomenon observed in previous experiments [26]. After being immersed in molten salts for three months, despite experiencing clear corrosion damage, all samples retained their structural integrity and did not suffer any fractures. Moreover, no deterioration due to pitting was observed in the samples, which was verified after polishing the samples.

Table 5 shows the mass loss and corrosion rate for the molten salts in contact with the different tested metal alloys. Mass loss is a variable that was quantified for the five alloys that were immersed in both molten salts. The results of mass loss obtained for the samples that were immersed in the Li_2_CO_3_-Na_2_CO_3_-K_2_CO_3_-LiOH∙H_2_O (56.65-12.19-26.66-4.51wt.%) molten salt showed that, in the case of half-immersed samples, Alloy 214 and Alloy 601 exhibited the highest mass loss; meanwhile, Alloy 625 and Alloy X exhibited the lowest mass loss. In contrast, in the case of completely immersed samples, Alloy 625 and Alloy 600 exhibited the largest mass loss; meanwhile, Alloy 214 and Alloy 601 exhibited the lowest mass loss. Furthermore, the results obtained for the samples that were immersed in the molten Li_2_CO_3_ showed that, in the case of half-immersed samples, Alloy 601 and Alloy 214 exhibited the highest mass loss; meanwhile, Alloy 625 and Alloy 600 exhibited the lowest mass loss. Moreover, in the case of completely immersed samples, Alloy 601 and Alloy X exhibited the largest mass loss; meanwhile, Alloy 600 and Alloy 625 exhibited the lowest mass loss.

Corrosion rate assessment is essential in the evaluation of the useful life of structures. It is also a key parameter to consider in the selection of materials for different settings and the types of corrosion inhibitors to be applied. For the five metals examined in this study, very similar corrosion rate values were obtained in the tests with both molten salts. Moreover, the corrosion rate trend was repeated in all cases. At the beginning (after one week of immersion) the corrosion rate values were the highest. However, in the second period of the experiment (after one month of immersion), the corrosion rate decreased significantly, and in the final stage (between one month and three months), the corrosion rate stabilized with a tendency to decrease. This trend can be attributed to the fact that at the beginning, the first corrosion products are formed and the samples experience a significant loss of mass due to corrosion deterioration. However, in the following stages, the oxides in the samples are passivated and act as protection for the metal.

Within the five metals examined in contact with both molten salts, the results of corrosion rate obtained for the samples that were immersed in the Li_2_CO_3_-Na_2_CO_3_-K_2_CO_3_-LiOH∙H_2_O (56.65-12.19-26.66-4.51wt.%) molten salt were similar to the ones obtained for mass loss in the case of half-immersed samples, among which Alloy 214 and Alloy 601 exhibited the highest corrosion rate; meanwhile, Alloy 625 and Alloy X exhibited the lowest corrosion rate. In contrast, in the case of completely immersed samples, Alloy 625 and Alloy 600 exhibited the largest corrosion rate; meanwhile, Alloy 214 and Alloy 601 exhibited the lowest rate. Furthermore, the results obtained for the samples that were immersed in the molten Li_2_CO_3_ showed that, in the case of half-immersed samples, Alloy 601 and Alloy 214 exhibited the highest corrosion rate; meanwhile, Alloy 625 and Alloy 600 exhibited the lowest corrosion rate. Moreover, in the case of completely immersed samples, Alloy 214 and Alloy X1 exhibited the largest corrosion rate; meanwhile, Alloy 600 and Alloy 625 exhibited the lowest corrosion rate. According to the literature [9], chromium and nickel oxides are products formed during the corrosion process and act as protection (passivated oxides) in the alloys. This corresponds with the fact that the lowest corrosion rate values were found for the alloys with the highest chromium and nickel concentrations of the five metals examined.

## 4. Conclusions

This study presents an evaluation of the corrosion behavior of two different carbonate-based molten salts, a Li_2_CO_3_-Na_2_CO_3_-K_2_CO_3_-LiOH∙H_2_O (56.65-12.19-26.66-4.51wt.%) mixture and a Li_2_CO_3_ salt, considering both non-inert atmospheres. Five metal alloys were experimentally evaluated to quantify mass losses and corrosion rates, including Alloy 600, Alloy 601, Alloy 625, Alloy 214, and Alloy X1.

This study highlights the promising performance of five commercially available metal alloys tested as suitable materials for constructing containers that would be in contact with molten carbonates. These alloys have demonstrated good performance in industrial environments related to high-temperature corrosion, attributed to their high nickel and chromium contents, even in the absence of any corrosion inhibition techniques. This underscores the critical importance of proper container material selection, to ensure the durability and reliability of the system. Practically, the findings suggest that industries can confidently utilize these alloys to enhance the longevity of their systems, potentially reducing maintenance costs and downtime.

Corrosion is a crucial aspect of system design, making it essential to determine the appropriate molten salt–metal pairings. The corrosion tests in this study were conducted using the immersion method to assess static corrosion under atmospheric conditions (air atmosphere) at 780 °C. The results of this analysis indicated that the tested metal alloys, despite experiencing clear corrosive damage during the period of experimentation, all retained their structural integrity and did not suffer any fractures. Moreover, no deterioration due to pitting was observed in the samples, which creates exposed surfaces in the structures causing deterioration through corrosion and, therefore, fracture of the material.

The corrosion rate was also evaluated, and for the five metal alloys tested with both carbonate molten salts, corrosion rate values between 0.0009 mg/cm^2^·yr and 0.0089 mg/cm^2^·yr were found. In all cases, since the corrosion rate was lower than 0.2 mg/cm^2^∙yr, the metal alloys tested are recommended for long-term service (according to the guide for loss of mass by corrosion used in the industry [23]).

The Li_2_CO_3_-Na_2_CO_3_-K_2_CO_3_-LiOH∙H_2_O (56.65-12.19-26.66-4.51wt.%) salt mixture was shown to be more corrosive than Li_2_CO_3_. The corrosion rate values found for the salt mixture were between 0.0090 mg/cm^2^·yr and 0.0006 mg/cm^2^·yr; meanwhile, the corrosion rate values found for Li_2_CO_3_ were between 0.0019 mg/cm^2^·yr and 0.0002 mg/cm^2^·yr. These results can be explained considering the fact the molten carbonates can produce corrosive anions such as CO_2_^−3^, and O^−2^ which can react with alloy cations (e.g., Fe^+3^, Al^+3^, Cr^+3^) [9]; but, in the case of the Li_2_CO_3_-Na_2_CO_3_-K_2_CO_3_-LiOH∙H_2_O (56.65-12.19-26.66-4.51wt.%) salt mixture, since it contains LiOH∙H_2_O, it can also form another corrosive anion such as OH^−^.

Within the five exanimated alloys in contact with both molten salts, Alloy 625 and Alloy 600 showed the best corrosion resistance performance in contact with molten Li_2_CO_3_, while Alloy 625 and Alloy 214 showed the best corrosion resistance performance in contact with the Li_2_CO_3_-Na_2_CO_3_-K_2_CO_3_-LiOH∙H_2_O (56.65-12.19-26.66-4.51wt.%) salt mixture.

## Figures and Tables

**Table 1:** Corrosion data from the literature for molten carbonate mixtures (adapted and extended from [9]).

Molten Carbonate Salt Mixture (wt.%)	Alloy	Temperature (°C)	Time of Exposure (h)	Metallographic Thickness (µm)	Corrosion Rate (mm/year)	Corrosion Rate (mpy)	Reference
Na_2_CO_3_-Li_2_CO_3_-K_2_CO_3_ (33.4-32.1-34.5)	HR3C	700	2000	25.41	n.a.	n.a.	[10]
		800	2000	56.66	n.a.	n.a.	[11]
	In601	450	120	10.5	n.a.	n.a.	[12]
	In800H	600	24	n.a.	0.451	17.77	[13]
		750	---	---	1.08	42.55	[13]
	310SS	600	---	---	0.632	24.90	[13]
		750	---	---	1.53	60.28	[13]
	321SS	750	---	---	4.64	182.82	[13]
	347SS	750	---	---	2.36	92.98	[13]
	AFA-	750	---	---	1.75	68.95	[13]
	OC6	---	---	---	---	---	[13]
	OC4	650	1000	29–55	n.a.	1.4	[9]
	In625	750	---	---	2.60	102.44	[13]
	HR224	650	1000	45	n.a.	0.2	[9]
Na_2_CO_3_-Li_2_CO_3_-K_2_CO_3_ (33-32-35)	DS2205	500	1600	n.a.	n.a.	n.a.	[14]
	DS2507	500	1600	n.a.	n.a.	n.a.	[14]
	301LNSS	600	1000	n.a.	n.a.	n.a.	[15]
Na_2_CO_3_-Li_2_CO_3_-K_2_CO_3_ (34-33-33)	DMV310N	700	1000	17	n.a.	n.a.	[16]
		750	1000	45	n.a.	n.a.	[16]
	SZ 1183	700	1000	27	n.a.	n.a.	[16]
		750	1000	70	n.a.	n.a.	[16]
	In625	700	1000	48	n.a.	n.a.	[16]
		750	1000	78	n.a.	n.a.	[16]
	In617	700	1000	20	n.a.	n.a.	[16]
		750	1000	59	n.a.	n.a.	[16]
	Haynes230	700	1000	59	n.a.	n.a.	[16]
		750	1000	37	n.a.	n.a.	[16]
Na_2_CO_3_-Li_2_CO_3_-K_2_CO_3_ (35.1-10.2-54.5)	316SS	600	1440	n.a.	1.71	67.37	[17]
Na_2_CO_3_-Li_2_CO_3_-K_2_CO_3_ (31.2-15.5-53.3)	316SS	600	1440	n.a.	2.07	81.56	[17]
Na_2_CO_3_-Li_2_CO_3_-K_2_CO_3_ (38-33-29)	316SS	600	1440	n.a.	1.03	40.58	[17]
Na_2_CO_3_-K_2_CO_3_ (47.19-52.81)	316LSS	750 under Ar	3024	n.a.	9.5	n.a.	[18]
	347HSS	750 under Ar	3024	n.a.	0.98 ± 0.04	n.a.	[18]
	In800H	750 under Ar	3024	n.a.	0.56 ± 0.025	n.a.	[18]
Li_2_CO_3_-K_2_CO_3_ (28-72)	310SS	600	500	n.a.	0.522	n.a.	[19]
	316LSS	600	500	n.a.	2.535	n.a.	[19]
	In625	600	500	n.a.	1.098	n.a.	[19]
Na_2_CO_3_-NaCl (59.4-40.6)	In625	650	768	n.a.	0.12–0.17	n.a.	[20]
	316LSS	650	768	n.a	2.0	n.a.	[21]

**Table 2:** Chemical composition of the metal alloys considered (data from manufacturer).

Element (%)	Alloy 600	Alloy 601	Alloy 625	Alloy X1	Alloy 214
Ni	72	58–63	58	40–50	74.64–69.61
Cr	14–17	21–25	20–23	20–23	15–17
Fe	6–10	18.39–7.69	5	17–20	2–4
Mn	1	1	0.50	1.00	<0.50
Cu	0.50	1	---	0.50	---
Si	0.50	0.50	0.50	1.00	<0.20
C	0.15	0.10	0.10	0.05–0.15	<0.05
S	0.01	0.01	0.01	0.03	<0.02
Al	---	1.00–1.70	---	0.50	4–5
Mo	---	---	8–10	8–10	<0.50
Nb + Ta	---	---	3.15–4.15	---	---
Co	---	---	1	0.50–2.50	<2.0
Ti	---	---	0.40	0.15	<0.50
Ag	---	---	0.40	---	---
P	---	---	0.01	0.04	<0.02
W	---	---	---	0.20–1.00	<0.50
B	---	---	---	0.01	<0.01
Y	---	---	---	---	0.01–0.04
Zr	---	---	---	---	<0.50

**Table 3:** Visual inspection of metal alloy samples after testing with Li_2_CO_3_-Na_2_CO_3_-K_2_CO_3_-LiOH∙H_2_O (56.65-12.19-26.66-4.51wt.%).

Metal Alloy	Before Testing		After One Week of Testing				After One Month of Testing				After Three Months of Testing			
			Before Polishing		After Polishing		Before Polishing		After Polishing		Before Polishing		After Polishing	
Alloy 600	** 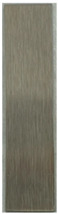 **	** 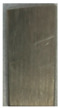 **	** 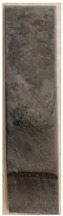 **	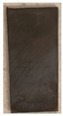	** 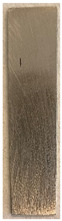 **	** 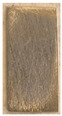 **	** 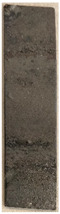 **	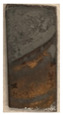	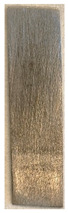	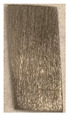	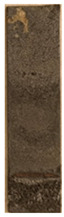	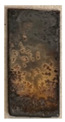	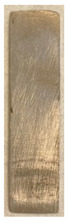	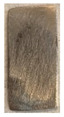
Alloy 601	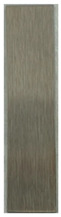	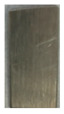	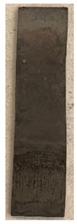	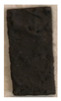	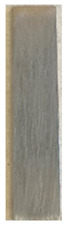	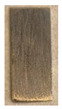	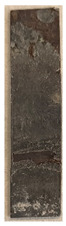	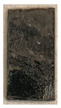	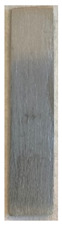	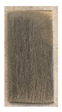	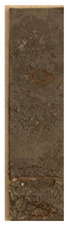	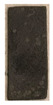	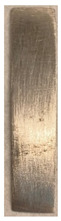	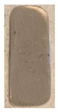
Alloy 625	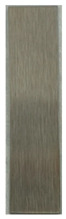	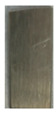	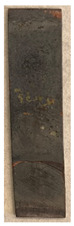	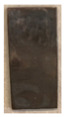	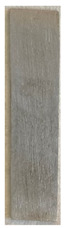	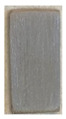	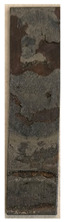	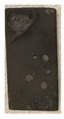	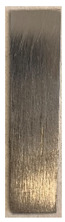	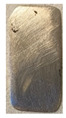	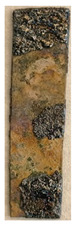	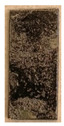	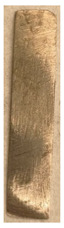	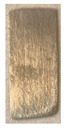
Alloy X1	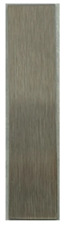	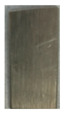	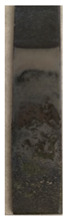	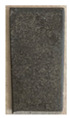	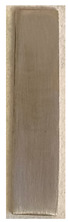	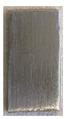	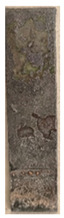	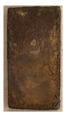	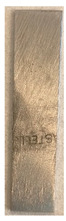	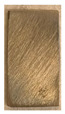	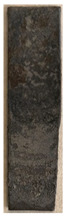	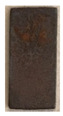	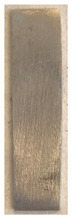	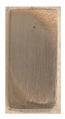
Alloy 214	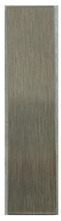	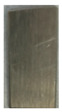	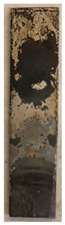	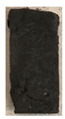	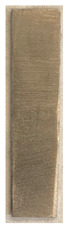	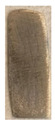	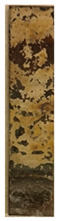	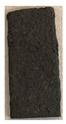	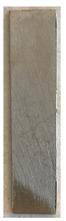	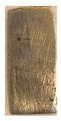	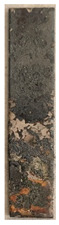	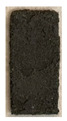	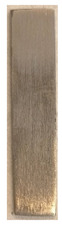	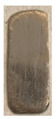

**Table 4:** Visual inspection of metal alloy samples after testing with Li_2_CO_3_.

Metal Alloy	Before Testing		After One Week of Testing				After One Month of Testing				After Three Months of Testing			
			Before Polishing		After Polishing		Before Polishing		After Polishing		Before Polishing		After Polishing	
Alloy 600	** 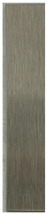 **	** 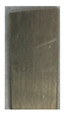 **	** 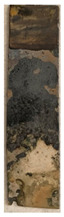 **	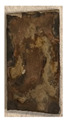	** 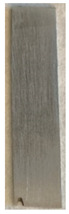 **	** 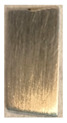 **	** 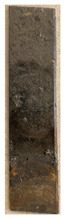 **	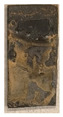	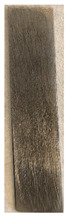	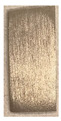	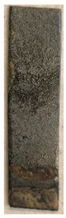	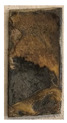	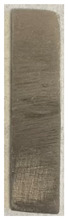	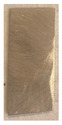
Alloy 601	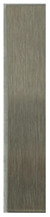	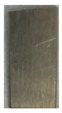	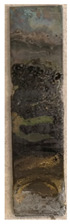	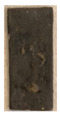	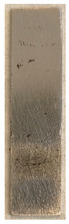	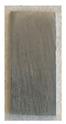	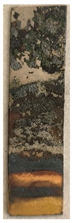	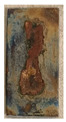	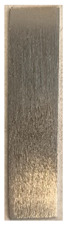	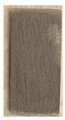	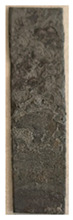	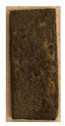	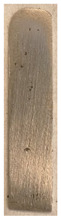	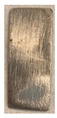
Alloy 625	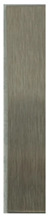	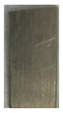	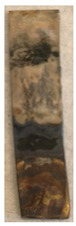	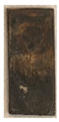	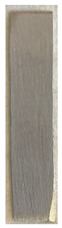	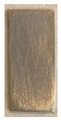	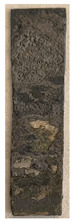	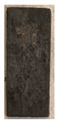	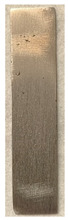	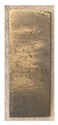	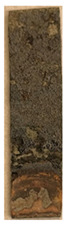	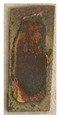	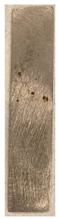	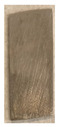
Alloy X1	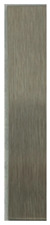	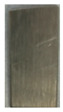	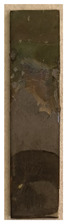	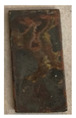	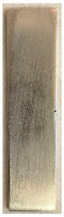	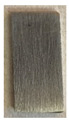	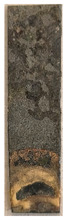	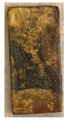	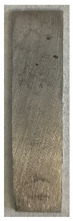	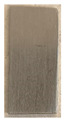	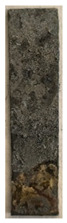	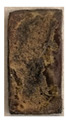	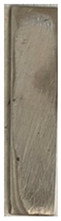	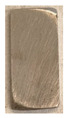
Alloy 214	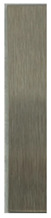	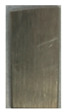	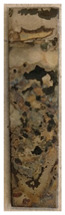	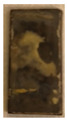	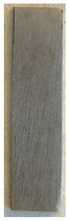	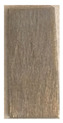	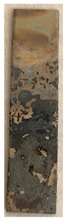	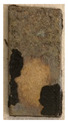	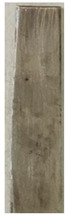	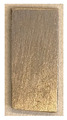	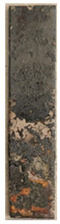	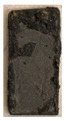	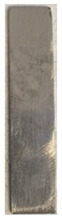	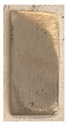

**Table 5:** Results of the corrosion tests.

Corrosion Test Procedure	Mass Loss	Corrosion Rate	
		mg/cm^2^·yr	mm/yr
Li_2_CO_3_-Na_2_CO_3_-K_2_CO_3_-LiOH∙H_2_O (56.65-12.19-26.66-4.51wt.%)			
Samples completely immersed	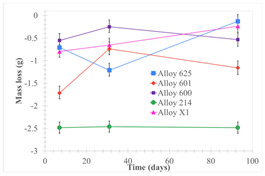	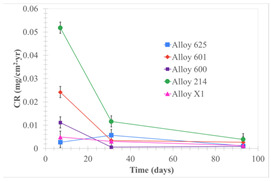	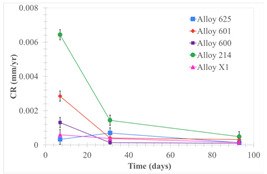
Samples half immersed	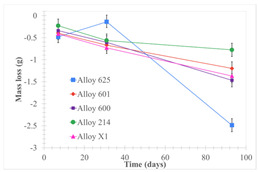	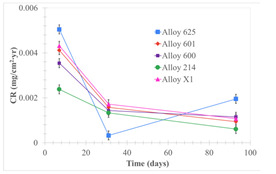	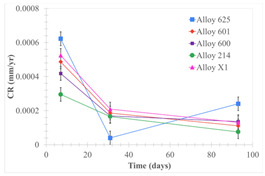
Li_2_CO_3_			
Samples completely immersed	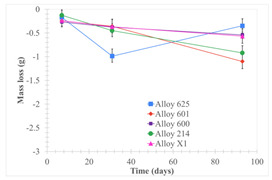	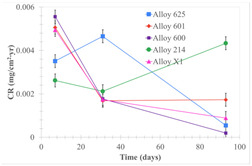	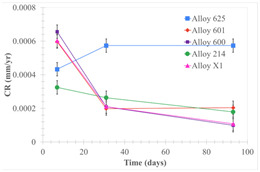
Samples half immersed	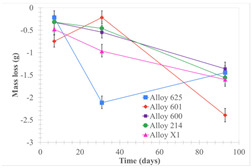	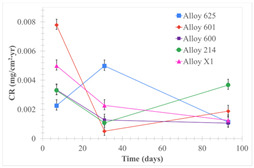	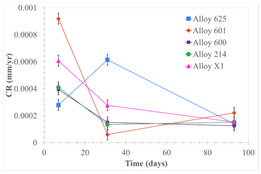

Solid points: experimental values. Error bar: overall measurement uncertainty.

## Data Availability

The original contributions presented in this study are included in the article. Further inquiries can be directed to the corresponding author.
